# Data: WireFishing-M: A multimodal dataset for deformable cable insertion using tactile, visual, and proprioceptive sensing

**DOI:** 10.1016/j.dib.2025.112136

**Published:** 2025-10-09

**Authors:** Tianyu Zhou, Hengxu You, Fang Xu, Jing Du

**Affiliations:** The Informatics, Cobots and Intelligent Construction (ICIC) Lab, University of Florida, 1949 Stadium Rd, Gainesville, FL 32611, USA

**Keywords:** Deformable object manipulation, Tactile sensing, Multimodal dataset, Robotic wire insertion

## Abstract

This article introduces WireFishing-M, a multimodal dataset designed to support research in deformable object manipulation, tactile sensing, and contact-rich robotic tasks. The dataset captures a robotic wire insertion scenario, where a Franka Emika Panda 7-DOF robotic manipulator equipped with an Allegro Robot Hand and a DIGIT tactile sensor performs repeated cable insertions into a transparent l-shaped PVC pipe (1″ Sch 40 NSF-61). The dataset includes seven different types of cables varying in physical properties. For each cable, we collected synchronized GelSight tactile images, multi-view RGB videos (front view, bottom view, and a side view monitoring the pipe opening for insertion outcome), end-effector poses, robot joint states, and externally estimated forces at the end-effector. The tactile sensor continuously captures contact interactions as the robot grips, inserts, and exits the cable from the pipe. WireFishing-M enables the development and benchmarking of multimodal models for perception, force estimation, manipulation policy learning, and success detection in deformable object tasks. The complete dataset is publicly available via Harvard Dataverse and is organized to facilitate direct use in robotic learning and simulation frameworks.

Specifications Table

**Table:** 

Subject	Computer Sciences
Specific subject area	Deformable object manipulation, tactile sensing, multimodal robot learning
Type of data	Image, Raw.
Data collection	Using a Franka Emika Panda 7-DOF robotic arm equipped with an Allegro Robot Hand and a DIGIT tactile sensor to insert seven different types of cables into a transparent l-shaped PVC pipe. Synchronized data were recorded from tactile sensors, RGB cameras, robot joints, and internal force estimation.
Data source location	1949 Stadium Rd, Weil Hall 360, University of Florida, Gainesville, FL, United States.
Data accessibility	Repository name: Harvard Dataverse Data identification number: doi:10.7910/DVN/XOPUXY 10.7910/DVN/MUOJXI Direct URL to data: https://doi.org/10.7910/DVN/XOPUXY https://doi.org/10.7910/DVN/MUOJXI
Related research article	None.

## 1 Value of the Data

• This dataset provides one of the first multimodal benchmarks for deformable cable insertion tasks, combining tactile, visual, proprioceptive, and force sensing. It enables research on manipulation strategies for handling flexible objects in contact-rich environments, where high-fidelity sensor feedback is essential.
• The data can be reused by researchers developing machine learning models for perception, control, and planning in robotics. In particular, it supports studies on contact detection, force estimation, material interaction modeling, and end-to-end learning for deformable object manipulation.
• The synchronized tactile (DIGIT), RGB video (multi-view), robot joint state, pose, and internal force data offer a comprehensive multimodal sensor stream for benchmarking sensor fusion methods. These data streams are aligned and time-stamped to allow exploration of both early and late sensor fusion architectures.
• Researchers in tactile sensing and hardware-in-the-loop simulation can use this dataset to develop or validate models of physical interaction. The real-world tactile image sequences provide rich ground truth data for contact localization, slip detection, and modeling deformation.
• This dataset supports the development of success detection models for robotic wire insertion tasks. With side-view video footage showing insertion success or failure, it can be used for training classifiers or reinforcement learning reward models for task completion monitoring.
• It is also valuable for researchers exploring generalization across object types. With seven types of cables varying in material and flexibility, the dataset enables studies on transfer learning and domain adaptation in deformable manipulation.

## 2 Background

The ability to manipulate deformable linear objects (DLOs), such as cables and wires, remains a significant challenge in robotic manipulation due to their high degrees of freedom, unpredictable behaviour, and sensitivity to contact interactions [1,2]. Traditional manipulation strategies often focus on rigid body dynamics, while tasks involving deformable objects require real-time sensing and adaptive control [3,4]. To address this challenge, our work focuses on the wire fishing task, a representative scenario in construction, electrical assembly, and robotics, where a cable must be inserted through a narrow, curved conduit [5,6].

To support research on this class of problems, we developed a robotic system using a Franka Emika Panda manipulator equipped with an Allegro Robot Hand and a DIGIT tactile sensor [7]. The system performs repeated insertion trials using cables of varying material properties. By collecting synchronized multimodal data during this process, we aim to provide a resource for advancing perception, learning, and control strategies for deformable object manipulation. This dataset may also support future modelling and benchmarking efforts in tactile sensing and multimodal fusion.

## 3 Data Description

The WireFishing-M dataset is organized into top-level folders representing individual cable types and insertion conditions. Each folder contains subfolders labeled by the date of data collection, and within each date folder are multiple trial folders. The trial numbers are used for file organization only and do not correspond to specific experimental variations.

Cable_(number)_Human/: Trials in which the robot hand was manually controlled by a human for cable insertion. These trials typically exhibit a higher success rate and smoother manipulation.

Cable_(number)_Random/: Trials in which the robot performed insertions from randomized initial positions and orientations, resulting in greater variability in outcomes.

Each trial folder contains the following five subfolders: global_view/: Bottom-view RGB images (.jpg) captured from a camera placed underneath the robot’s workspace. inner_view/: Side-view RGB images (.jpg) focused on the pipe opening, used to visually assess insertion success or failure. local_view/: Front-view RGB images (.jpg) showing the robot’s approach and interaction with the cable. tactile_view/: Grayscale tactile images (.jpg) from the DIGIT sensor. Each image is vertically stacked with contact views from three fingers: index (top), thumb (middle), and middle (bottom). robot_data/: A NumPy .npy file containing time-series robot data with the following structure:

[0–2]: End-effector force (Fx, Fy, Fz)

[3–5]: End-effector torque (Tx, Ty, Tz)

[6–11]: End-effector pose (position x, y, z and orientation quaternion x, y, z, w)

[12–18]: Joint angles of the 7-DOF Franka Emika Panda robot

[19]: Task outcome (1 = success, 0 = failure)

Each trial folder contains several thousand frames recorded during a single cable insertion attempt. The data streams are timestamp-aligned and include both human-guided and randomized trials.

**Fig. 1:**
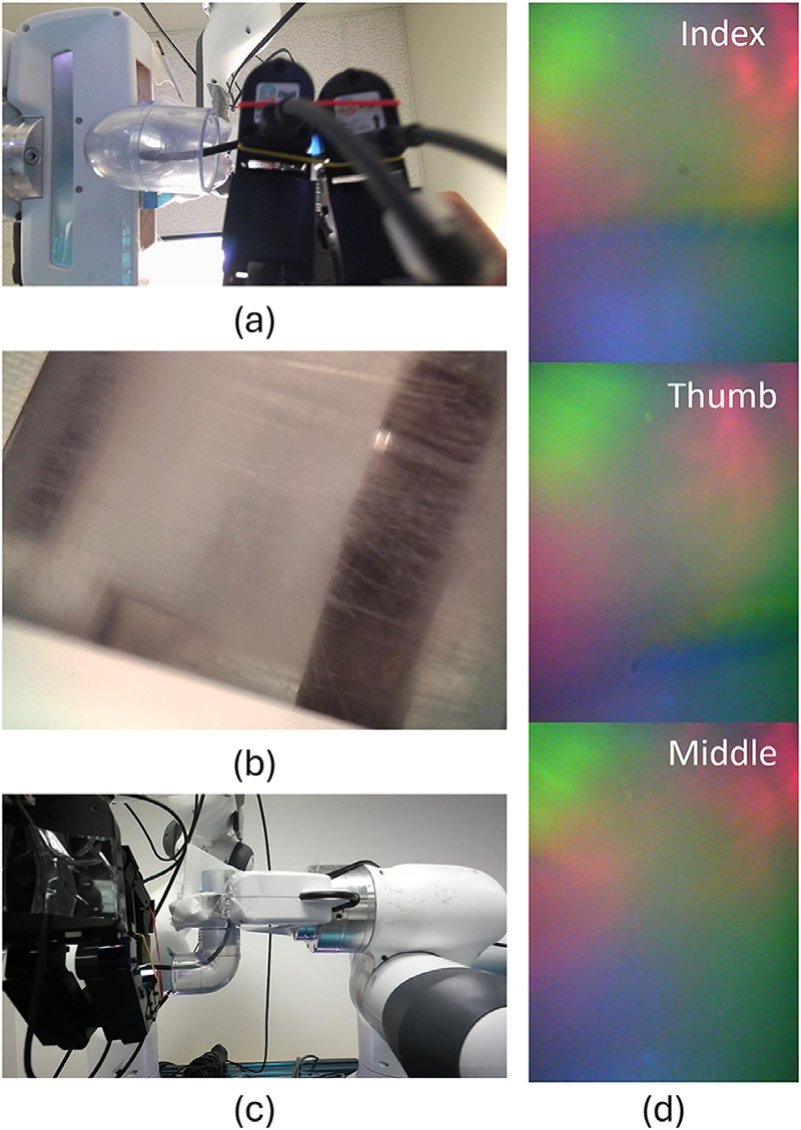
Example data sample. (a) Bottom view, (b) Side view, (c) Front view, (d) Tactile image.

As illustrated in Fig. 1, the dataset includes synchronized multimodal visual and tactile observations from various viewpoints. Fig. 1.a shows the bottom view captured from the global_view camera, capturing detailed interactions between the robot hand and the cable. Fig. 1.b presents the side view from the inner_view camera, focused on the pipe opening to assess whether the cable was successfully inserted. Fig. 1.c displays the front view from the local_view camera, providing an overview of the workspace and cable trajectory. Fig. 1.d shows a tactile image from the tactile_view folder, recorded by the DIGIT GelSight sensor. Each image encodes contact deformation from three fingertips, ordered from top to bottom as index, thumb, and middle finger. These views collectively provide a comprehensive visual and tactile record of each manipulation trial, enabling multi-perspective analysis of cable behavior and contact dynamics.

Table 1 provides a detailed summary of the cable types used in the dataset, along with sample images, physical descriptions, and the number of trials collected under two different conditions: Random Manipulation Data, in which the robot inserted the cable with randomized position and orientation, and Human Demo Data, in which the robot hand was manually guided by a human to perform the insertion.

**Table 1:** Overview of Cable Types and Corresponding Trial Counts in the WireFishing-M Dataset.

Cable Number	Cable Type	Description	Random Manipulation Data	Human Demo Data
1		Flexible, Thin, Lightweight, Braided nylon cover	∼2900,000	5962
2		Rigid, Thick, Heavy-duty, No cover	84,621	5994
3		Rigid, Thin, Lightweight, No cover	57,285	6668
4		Less Flexible, Thin, Lightweight, Braided nylon cover	169,602	6170
5		Less Rigid, Thin, Lightweight, No cover	284,262	7624
6		Rigid, Thin, Lightweight, Braided nylon cover	27,232	4949
7		Flexible, Thin, Lightweight, No cover	384,475	7141

	Total		∼3907,477	44,288

Seven cables with varying degrees of rigidity, flexibility, cover texture, and thickness were selected to represent a diverse range of real-world wiring scenarios. The dataset includes a large number of frames across conditions, with over 3.9 million frames collected in randomized trials and 44,288 frames in human demonstrations. This distribution supports research on generalization, robustness, and imitation learning for deformable object manipulation tasks.

## 4 Experimental Design, Materials and Methods

### 4.1 Robotic system and hardware setup

The WireFishing-M dataset was collected using a custom-designed robotic manipulation platform (Vention Inc.) built to simulate realistic deformable object insertion tasks. The system is centered around a Franka Emika Panda 7-DOF robotic manipulator mounted on the workstation. An Allegro Robot Hand (Shadow Robot Company) with four fingers and 16 degrees of freedom is attached to the robot’s end-effector flange. A DIGIT tactile sensor (GelSight Inc.) is mounted on the index, middle, and thumb fingertips, capturing high-resolution images of contact deformation during grasping and insertion. The three streams are concatenated into a single tactile image of 240×960 pixels at 5 fps.

To provide external visual perspectives, multiple cameras were integrated:

• Front camera: Femto Mega RGB-D camera, positioned ∼18.5 inches above the platform and ∼20 inches from the pipe, capturing global views of the robot, pipe, and cable. RGB images are recorded at 1280×720 pixels, 5 fps.
• Bottom camera: Microsoft Azure Kinect DK, positioned ∼7 inches above the platform, recording local RGB views of the cable insertion region at 1280×720 pixels, 5 fps.
• Pipe camera: 16.4 ft USB Endoscope Camera with integrated LED illumination, outside the transparent PVC pipe to capture pipe opening, used to visually assess insertion success or failure. Images are recorded at 640×480 pixels, 5 fps.

The insertion target is a transparent l-shaped PVC pipe assembly: the l-shaped pipe has an inner diameter (ID) of 1.3 inches and an outer diameter (OD) of 1.6 inches; the straight pipe connected on top has 1.0-inch ID and 1.3-inch OD, extending 2 inches above the l-shaped pipe. The full pipe assembly stands ∼18.5 inches above the platform surface. Seven types of flexible cables with different stiffness, surface textures, and diameters were used to ensure diversity in contact dynamics.

The robot operates under impedance control mode, and all data streams (robot joint states, end-effector poses, tactile images, camera images) are collected at 5 fps. Synchronization across sensing modalities is achieved via ROS topics with timestamp alignment.

### 4.2 Sensor configuration and calibration

To capture the cable insertion process from multiple perspectives, three RGB cameras were positioned around the robot workspace: A front-view camera, facing the robot, captures the interaction between the robot hand and the cable. A bottom-view camera, placed beneath a transparent working surface, provides an overhead view of the cable’s motion path. A pipe-side camera, positioned laterally near the pipe opening, is used to monitor and assess insertion outcomes (success or failure).

A DIGIT tactile sensor was mounted on one of the Allegro fingers to capture high-resolution images of surface deformation during grasping and manipulation. The DIGIT sensor records at approximately 30 frames per second, providing rich contact information for each trial.

Robot state data, including joint angles, end-effector pose, and externally estimated force and torque, were acquired using the Franka Emika Panda ROS interface, specifically from the /franka_state_controller/franka_states topic. The end-effector pose was computed via forward kinematics based on the robot’s internal joint state.

All sensor streams were timestamped and recorded through a central ROS node. Data synchronization was performed offline based on software timestamps. No hardware-level synchronization was applied. The following calibration steps were performed: Camera calibration: Both intrinsic and extrinsic parameters were calibrated using a standard checkerboard pattern. DIGIT sensor calibration: Performed according to the manufacturer’s procedures to ensure consistent grayscale intensity and image alignment across sessions.

### 4.3 Task description and data collection procedure

Each trial begins with the robot grasping a cable segment at a predefined location. The Allegro hand is programmed to maintain a stable grip throughout the task using fixed joint commands. The robot then attempts to insert the cable into a transparent l-shaped PVC pipe mounted on the workspace. To introduce variability and simulate diverse manipulation scenarios, each cable insertion is segmented into a series of discrete motion steps. At the start of each trial, the robot moves to a randomized initial pose. Then, during each motion step: The end-effector move forward to the pipe along the x-axis by a random distance sampled from a uniform range of 0 to 2 cm. Simultaneous small adjustment are applied in the y- and z-axes, sampled from –1 to 1 cm. Random orientation is applied in pitch and yaw, each drawn from a range of –15° to 15°

If the cumulative forward displacement exceeds a predefined insertion depth, the robot resets to a new random starting pose and repeats the process. This open-loop strategy enables the collection of varied contact conditions, including successful insertions, partial insertions, misalignments, and collisions. All robot motions were executed using impedance control via the native Franka controller, allowing compliant interactions with the cable and pipe during contact.

To determine the outcome of each insertion attempt, the pipe-side RGB camera image was converted to grayscale, and the number of dark (near-black) pixels in a predefined region near the pipe outlet was counted. If the number of pixels below a grayscale threshold of intensity < 50 exceeded 40 pixels, the trial was automatically labeled as a successful insertion. Otherwise, it was labeled as a failure. This automated labeling procedure ensured consistent and scalable annotation across thousands of trials.

Both randomized robot insertion trials and human-guided demonstrations are included in the dataset. In human demonstration trials, humans handle the robot end-effector to mimic expert-level insertion behavior, resulting in higher success rates and smoother trajectories.

**Fig. 2:**
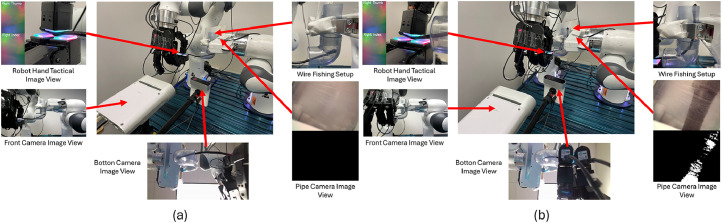
Multimodal setup and visual feedback during the wire insertion task (Franka arm, Allegro hand, DIGIT, pipe, cameras). (a) Example initial pose. (b) Example ending pose.

Fig. 2.a shows the system configuration at the initial stage of cable insertion. The center image presents the overall experimental setup, including the Franka Emika Panda arm, Allegro Robot Hand, DIGIT tactile sensor, and camera placements. The upper left inset displays a close-up of the DIGIT sensor and an example RGB tactile image. The bottom left and bottom middle show the front and bottom RGB camera views, respectively. The upper right image provides a side view of the cable inside the transparent l-shaped pipe. The bottom right inset shows the pipe-side camera view, along with the corresponding grayscale image used for automatic insertion success detection. Fig. 2.b displays the same views during a successful cable insertion. Compared to (a), the bottom right grayscale image in (b) contains a significant number of dark pixels near the pipe exit, which are used as indicators of successful insertion in the automated labeling algorithm.

### 4.4 Software stack and data recording

All experiments were conducted using the Robot Operating System (ROS) Melodic, running on Ubuntu 18.04. The entire robotic system, including the Franka Emika Panda arm, Allegro Robot Hand, DIGIT tactile sensor, and three RGB cameras, was integrated and controlled through ROS nodes. Custom Python scripts were developed to coordinate sensor control, robot motion, and data recording.

Data acquisition was fully implemented in Python, leveraging the ROS interface to: Control and execute impedance-based trajectories on the Franka arm. Maintain fixed grasping configurations for the Allegro hand. Capture and store synchronized RGB images from the front, bottom, and pipe-side cameras. Record high-resolution tactile images from the DIGIT sensor at approximately 5 FPS. Extract robot joint states, end-effector pose, and internal force/torque estimates from the /franka_state_controller/franka_states ROS topic.

All data streams were timestamped and saved during each trial. After each session, the raw data was post-processed into structured folders. Images were saved in .jpg format, while robot state data was saved in NumPy array (.npy) format with a consistent structure for ease of use in downstream machine learning pipelines.

## Limitations

While the WireFishing-M dataset provides valuable multimodal data for deformable object manipulation, there are several limitations to consider:

• **Limited diversity of object types**: The dataset includes only seven types of cables, which limits the generalizability of models trained solely on this data to a broader range of deformable linear objects.
• **Fixed environment and insertion setup**: All trials were performed using the same l-shaped PVC pipe in a controlled laboratory setting. Variations in pipe geometry or environmental conditions are not captured.
• **Sensor noise and desynchronization**: Although timestamps were logged for all data streams, slight misalignment may occur due to asynchronous sensor readouts and variable framerates, especially for the cameras and DIGIT sensor.
• **Binary success labels**: Insertion outcomes were labeled manually as success or failure using visual inspection, without fine-grained annotations of failure modes or partial insertions.

These limitations should be taken into account when applying the dataset to downstream tasks or model evaluation.

## Ethics Statement

The authors have read and follow the ethical requirements for publication in Data in Brief and confirming that the current work does not involve human subjects, animal experiments, or any data collected from social media platforms.

## Credit Author Statement

**Tianyu Zhou**: Methodology, Software, Data Curation, Writing - Original Draft. **Hengxu You**: Validation, Resources, Writing - Review & Editing. **Fang Xu**: Validation, Investigation, Data curation. **Jing Du**: Conceptualization, Writing - Review & Editing, Supervision.

## Data Availability

Harvard DataverseWireFishing-M-2 (Original data)
Harvard DataverseWireFishing-M-1 (Original data)
